# Interfaces with the peripheral nervous system for the control of a neuroprosthetic limb: a review

**DOI:** 10.1186/s12984-020-00667-5

**Published:** 2020-03-10

**Authors:** Kadir A. Yildiz, Alexander Y. Shin, Kenton R. Kaufman

**Affiliations:** 1 Motion Analysis Laboratory, Department of Orthopedic Surgery, Mayo Clinic, Rochester, MN USA; 2 Department of Orthopedic Surgery, Mayo Clinic, Rochester, MN USA; 3 Motion Analysis Laboratory, W. Hall Wendel, Jr., Musculoskeletal Research, 200 First Street SW, Rochester, MN 55905 USA

**Keywords:** amputation, artificial limb, prostheses and implants, neuroprosthesis, peripheral nervous system, neural conduction, electromyography, electric stimulation, electrodes, extremities

## Abstract

The field of prosthetics has been evolving and advancing over the past decade, as patients with missing extremities are expecting to control their prostheses in as normal a way as possible. Scientists have attempted to satisfy this expectation by designing a connection between the nervous system of the patient and the prosthetic limb, creating the field of neuroprosthetics. In this paper, we broadly review the techniques used to bridge the patient’s peripheral nervous system to a prosthetic limb. First, we describe the electrical methods including myoelectric systems, surgical innovations and the role of nerve electrodes. We then describe non-electrical methods used alone or in combination with electrical methods. Design concerns from an engineering point of view are explored, and novel improvements to obtain a more stable interface are described. Finally, a critique of the methods with respect to their long-term impacts is provided. In this review, nerve electrodes are found to be one of the most promising interfaces in the future for intuitive user control. Clinical trials with larger patient populations, and for longer periods of time for certain interfaces, will help to evaluate the clinical application of nerve electrodes.

## Background

The earliest example of a prosthetic limb comes from the ancient Egypt in the 15^th^ century BC; it is a simple hallux prosthesis for the right foot, manufactured from leather and wood [1]. Since this earliest example, there has been a considerable number of prosthetic limb designs. Some were worn for cosmetic purposes, with very little function [2]. While body-powered prosthetics remain popular today, they are designed with a functional intent and do not confer intuitive control. The lack of intuitive control prompted the development of neuroprosthetic interfaces, which brought the potential of natural use of the artificial limbs by the user’s own nervous system. As the name implies, these interfaces aim to communicate with the user’s nervous system. The nervous system is composed of a central and peripheral system. Communication and interfacing with the two systems have been described [3, 4]. Interfacing with the peripheral nervous system (PNS) will be the focus of this review.

There is a substantial body of literature regarding neuroprosthetic interfaces. Comprehensive reviews on related topics such as neural control of movement [5], brain-computer interfaces [6], and targeted muscle reinnervation [7] exist. There is also a great amount of attention on developing dexterous prosthetic limbs. However, the information on specifically interfacing the PNS to control these advanced prostheses is more sparse. The relevant reviews either concentrate on a certain aspect of the field such as nerve electrodes [8], myoelectric control [9], cuff electrodes [10], or  intra-fascicular electrodes [11]; or can be considered outdated [12] because of recent advances in the field. This paper addresses this knowledge gap with a broad review of current PNS interface methods.

This review comprises both studies that were encountered through search in digital literature and the relevant journals in Mayo Clinic Library. Digital libraries such as PubMed, IEEE Xplore, and ScienceDirect were used. The search keywords were “neuroprosthesis”, “myoelectric prosthesis”, “peripheral nerve interface”, “neuroprosthetic control”, “extra-neural electrode”, “inter-fascicular electrode”, “intra-fascicular electrode”, and “regenerative electrode”. Specific journals such as Journal of Neuroengineering and Rehabilitation, Journal of Neural Engineering, and Proceedings of the IEEE were explored for specific keywords. The inclusion criteria were:

- Studies that present a new interfacing method
- Clinical or animal studies that test a specific aspect of an interfacing method
- Clinical or animal studies that combine two or more different interfacing methods
- Studies that define a new classification algorithm

In this review, we present a broad overview of the interfaces with the PNS. We commence by giving a brief description of the PNS and continue by describing both the electrical and non-electrical interfaces, followed by delineating the considerations regarding the interface design. We then lay out improvements in fabrication technologies which can promote more desirable tissue response. We conclude by outlining the long-term outcomes of the interfaces and comparing them in terms of longevity.

## Brief description of the peripheral nervous system

The PNS comprises the cranial and spinal nerves including their roots, autonomic and sensory ganglia, glial cells, as well as the connective tissue elements. A peripheral nerve consists of numerous nerve fibers arranged in fascicles, supported by three coverings. The innermost endoneurium contains axons and their surrounding Schwann cells, collagen fibers, fibroblasts, capillaries, resident macrophages, and a few mast cells [13]. Each fascicle is surrounded by the perineurium which maintains the blood-nerve barrier and consists of separate layers of flattened perineurial cells together with sheets of collagen. The outermost epineurium is composed of moderately dense connective tissue along with blood vessels and lymphatic vessels which run longitudinally in the epineurium. The small blood vessels supplying the nerve, called vasa nervorum, arise from the branches of regional vessels and form a vascular plexus in the epineurium, pass through the perineurium and arrive at the endoneurium as capillaries. All three coverings contain the free ends of nervi nervorum, which are the small nerve filaments innervating the sheath of a larger nerve.

There are several ways to classify nerve fibers. They can be classified into three major groups depending on their conduction velocities and diameters. They can also be named as afferent or efferent based on the direction of their signals. The afferent fibers carry sensory signals to the central nervous system, while the efferent fibers carry motor signals to the effectors (gland, muscle). Each efferent fiber together with the muscle fibers that it innervates, constitute a motor unit. The controlled contraction of a muscle is achieved via coordination of its motor units.

Communication within the nervous system is primarily through the rate and pattern of action potentials, and it is the number or spacing of these action potentials per unit time that code information. Thus, information can be introduced into the nervous system by inducing action potentials, or these action potentials can be read to obtain information from the nervous system [14].

## Methods to interface the peripheral nervous system

### Electrical methods

Electrical stimulation of nerve and muscle, and recording of neural electrical activity are the basis of emerging prosthetic control. Stimulation is used to elicit depolarization of the membranes of the excitable cells (neurons, myocytes), and neural activity is recorded as action potentials by electrodes in the vicinity of the neurons [15]. A critical aspect of electrical stimulation is its higher level of safety risk compared to recording electrical activity, which can result in both electrode and tissue damage. The proposed mechanisms of stimulation-induced tissue injury include hyperactivity of neurons depleting metabolic fuels, changes of ionic concentrations across cell membranes, and excessive formation of toxic electrochemical reaction products creating free radicals [16]. It has been known for decades that electrical trauma to the nerves is dependent upon a few of the stimulation parameters, especially pulse frequency and pulse duration. Correspondingly, low stimulus frequencies with short durations, sufficient to provide the sought clinical response, should be applied in order to minimize nerve damage [17].

The specific methods to interface to the peripheral nerves include myoelectric systems and nerve electrodes. Muscle electrodes are the fundamental elements of myoelectric systems, and can be divided into two categories based on their invasiveness: surface electrodes that are placed on the skin, and implanted electrodes that are applied directly to the muscles. Implanted electrodes can be further subdivided into two types: epimysial electrodes which are sutured onto the surface of a muscle, and intramuscular electrodes which pierce the epimysium. Nerve electrodes can be classified as extraneural or intraneural according to their location with respect to the epineurium [12]. Intraneural electrodes that penetrate the epineurium but not the nerve fascicles are called inter-fascicular electrodes. Electrodes that penetrate both the nerve and the fascicles are called intra-fascicular electrodes. Specific electrodes that are designed to be placed transversely at the cut end of a transected nerve are named regenerative electrodes. An important characteristic of the nerve electrodes is that they pose a direct interface to the PNS, whereas myoelectric systems are primarily connected to the muscles leading to an indirect interface with the PNS.

#### Myoelectric systems

The most frequent way to interface to the PNS is to utilize the innervation of remaining muscle groups after an amputation. Originally, these muscle groups are not created to control the required action, rather they are aimed at executing different motor tasks. Accordingly, the user of a myoelectric prosthesis needs to relearn to perform certain actions through repetitive exercises, which usually takes many months.

There are several approaches to establish myoelectric systems, from simple non-invasive techniques to more advanced reconfiguration procedures. As these systems interface with the muscles, an additional feedback modality should also be employed in order to close the loop and provide the user with sensory feedback [18]. There are both non-invasive and invasive feedback systems. As the nerve electrodes directly interface with the peripheral nerves, they provide the opportunity to stimulate afferent axons to reproduce the sensations of the prosthesis user. Thus, nerve electrodes constitute an invasive sensory feedback system. Intuitive motor control can be attained via the use of the advanced techniques such as targeted muscle reinnervation (TMR), regenerative peripheral nerve interfaces (RPNIs) or the use of myoelectric pattern recognition [19–21].

The simplest method is to apply surface electrodes made of biocompatible metals on patient’s skin. The action potentials generated by the underlying muscles are recorded by these electrodes, and these recorded signals are detected, decomposed, and processed by various different methodologies [22], such as external software, for the prosthesis to perform an action. Thus, control of the prosthetic limb is dependent on the activation of residual muscles [23]. This non-invasive, uncomplicated method has certain drawbacks, such as the requirement of daily placement and calibration, the need for maintenance of the skin condition, movement artifacts, recording from unintended muscles, and low signal-to-noise ratio [24]. If the residual muscle mass of the extremity is inadequate, this method is unfavorable.

**Fig. 1:**
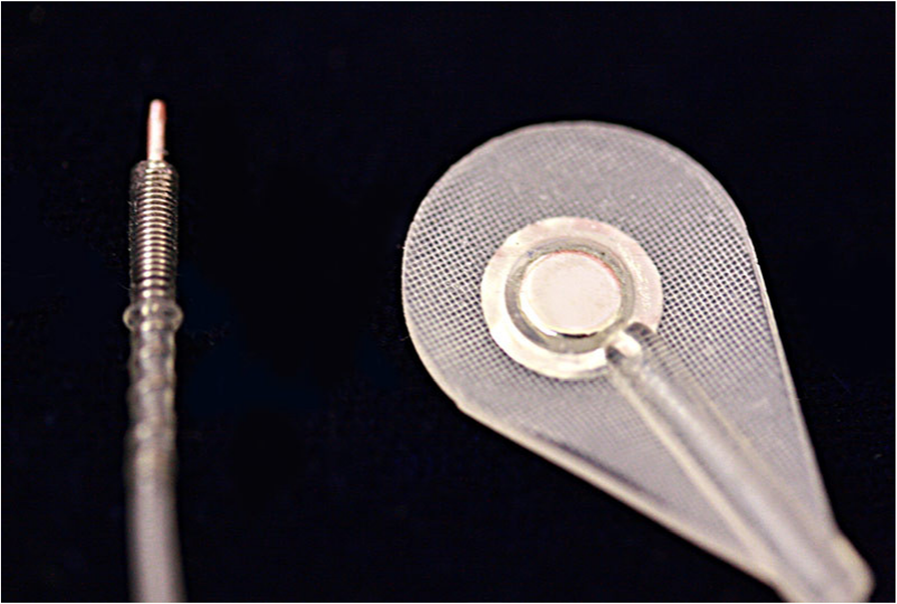
Epimysial electrode [25]

An alternative approach to record impulses and stimulate muscles is to utilize implantable muscle electrodes. Although invasive, this method provides increased spatial resolution and increased signal-to-noise ratio. It also eliminates the need for daily placement and issues with skin conditions. There are two approaches for implantable muscle electrodes: securing the electrode to the epimysium or implanting it intramuscularly. The less invasive one, the epimysial electrode (Fig. 1), can be sutured to the epimysium close to the motor endplate. The optimal place can be estimated via stimulation of the muscle intraoperatively [26]. One of the common designs consists of a platinum-iridium disc with silicone elastomer backing. It has been found suitable and reliable in both upper and lower extremity applications in various clinical studies [27, 28]. A recent clinical study, carried out with three transhumeral amputees with osseointegrated implants, compared the influence of surface electrodes and epimysial electrodes on grip force control and motor coordination [29]. Epimysial electrode implantation was found to enhance grip force control, while the subjects showed no improvement in motor coordination conceivably due to poor sensory feedback.

A prevalent model of an intramuscular electrode is composed of a coiled wire with an exposed uninsulated tip at one end. The wire is usually made up of stainless steel and insulated with Teflon. It is placed percutaneously using a guide wire. It has shown good mechanical performance for at least 24 months clinically, maintaining its position and having a low rate of breakage. Skin erythema at the place of injection was the most common complication, attributed to various etiologies [30]. A more recent design, the double-sided intramuscular electrode, incorporates a thin polyimide filament attached to a cannula for insertion. It contains 12 recording sites on the top side of the filament, and 3 stimulation sites on the bottom side, with an intermediate shielding layer made of platinum to prevent stimulation artifacts [31].

Aside from the use in prosthetic control, implanted muscle electrodes can also be used to re-establish trunk stability in patients with spinal cord injury, when implanted in the back and hip extensor muscles [32]. A 17-year follow up of two paraplegic patients with thoracic spine injury inspected the influence of stimulation of erector spinae, iliopsoas, and several lower extremity muscles via a system incorporating percutaneous muscle electrodes on walking ability [33]. The patients used the system at home to exercise. They were able to climb stairs, walk for 20 minutes, and stand for 18 to 20 minutes per session. Multiple complications occurred including localized infections, burns, lead breakage, and local rejection, leading to frequent electrode replacement, for which the authors conclude that the system complies with short term rehabilitation. On the other hand, femoral nerve stimulation with cuff electrodes were found to produce 300% longer standing times than vastus lateralis muscle stimulation via implanted muscle electrodes in a paraplegic patient, although the erector spinae and hip extensors were implanted with muscle electrodes in both occasions [34].

**Fig. 2:**
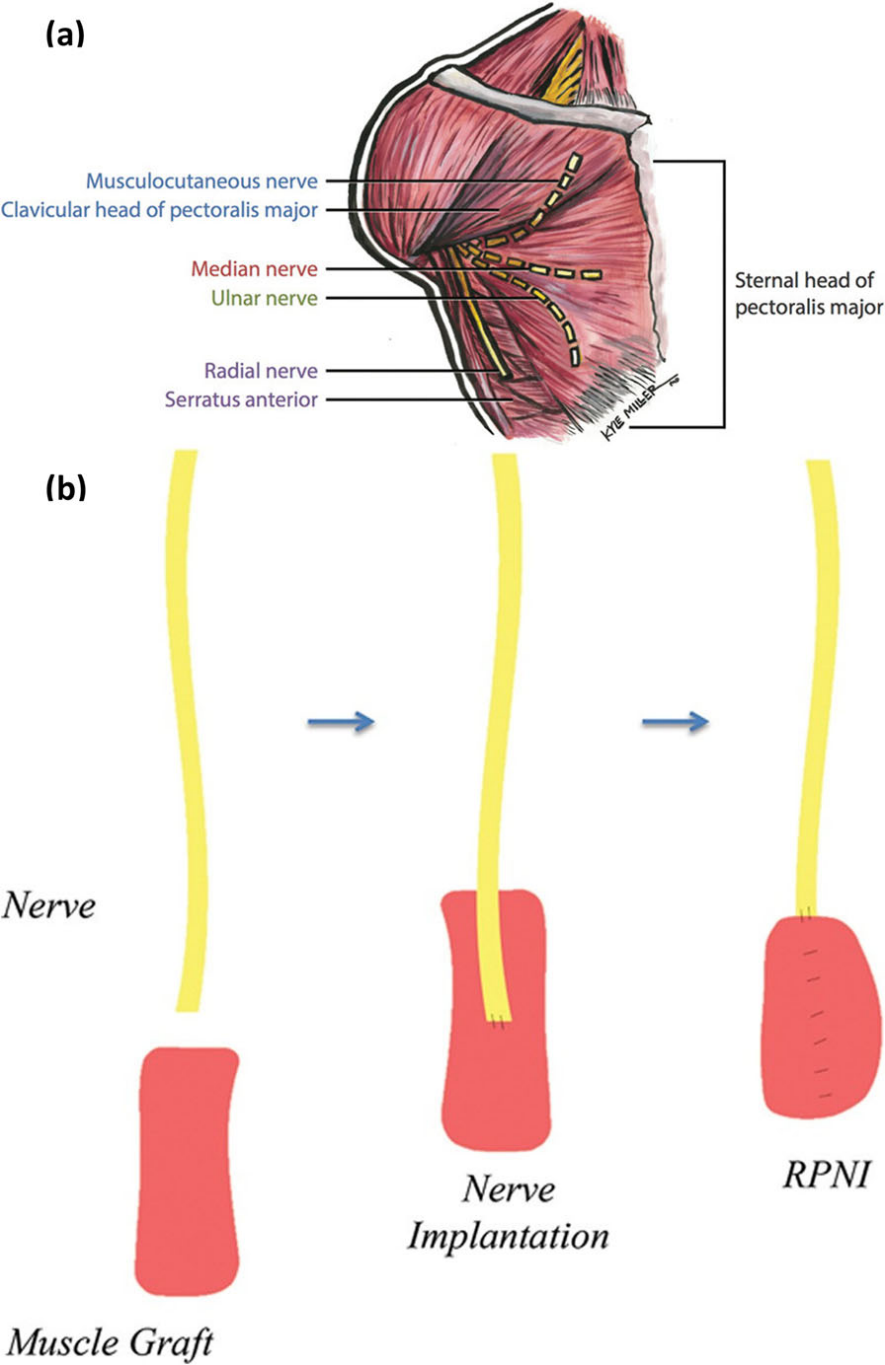
Myoelectric systems. **a** Surgical plan for the TMR for high amputations [36] **b** Illustration of RPNI construction [37]

All of the myoelectric systems described above depend on activation of the post-amputation residual muscle mass. An alternative approach is TMR, which redirects residual nerves to reinnervate new muscle targets. This method, first practiced successfully in a bilateral shoulder disarticulation amputee [35], consists of redirecting the severed nerves to new muscle targets. For instance, in an upper limb amputee, the median nerve is transferred to the sternal head of the pectoralis major muscle (Fig. 2a). Consequently, the patient’s desire to flex the wrist provokes action potentials and thus the generation of electromyography (EMG) signals in the sternal head of the pectoralis major muscle. During TMR surgery, all native motor innervation of the target muscle must be divided to prevent unwanted EMG signals confounding prosthesis control [38]. With this technique, the prosthetic control is more natural compared to conventional myoelectric methods, and the patients are more efficient in performing tasks [39]. TMR is also found to reduce phantom limb discomfort and neuroma pain after amputation [40]. The most crucial limiting factors arise from the fact that TMR is performed in conjunction with surface electrodes. Again, the disadvantages of surface electrodes exist. Therefore, the current research seeks to use TMR with implanted interfaces, such as the Implantable Myoelectric Sensor (IMES) [41].

Another option is to utilize RPNIs, which are surgically constructed from muscle grafts obtained from expendable skeletal muscle in the residual limb or from a distant site. These muscle grafts are neurotized by the terminal branches of the residual nerves (Fig. 2b), which are surgically dissected into its fascicles before the neurotization [19]. The most important advantage compared to TMR is that the operation is not limited to a certain anatomical area. When combined with epimysial electrodes, this mechanism showed good signal transduction and viability for seven months [42]. In another animal study, RPNIs were combined with intramuscular electrodes and demonstrated the chronic recording capability of this method for up to twenty months [43]. RPNIs have also shown promising results in the treatment of neuroma pain [37]. An important point to consider when RPNIs are going to be utilized with implanted muscle electrodes is the time of electrode placement. A recent study explored the effects of epimysial electrode installation and stimulation of deinnervated and devascularized free muscle grafts during the early recovery phase [44]. Histologic results showed an inflammatory state, and abnormal EMG signals with easier fatigability of the muscles, suggestive of myopathy. This study concludes that the muscle electrodes should be implanted 1-2 weeks after surgery to have time for early regeneration with adequate angiogenesis, and to enable biocompatible interfacing.

#### Extraneural electrodes

There are various different types of electrodes to directly interface with the peripheral nerves. Extraneural electrodes are the least invasive ones. There are four major types of extraneural electrodes: the epineural electrode, the helical electrode, the book electrode, and the cuff electrode.

Epineural electrodes are composed of a layer of insulation material, which contains one or more electrical contacts. These are sutured onto the epineurium. After the implantation procedure, fibrous tissue grows around the electrode as part of a foreign body reaction and this is profitable since it stabilizes the electrode. Following this stabilization process, signals from the nerves are both accurate and reproducible [37, 45]. These electrodes also affect muscle fiber distribution. In a 26-week animal study, 5 epineural electrodes were implanted unilaterally in 6 sheep to stimulate the lower extremity muscles. Muscle biopsies at the end showed increased type I muscle fiber distribution compared to the contralateral side, with no change in type II fiber distribution [46]. A clinical study compared the epineural electrodes and intramuscular electrodes in patients who had anal neosphincter reconstruction with graciloplasty [47]. The voltage required to stimulate the sphincter was lower in patients with epineural electrodes. However, 26% of the patients in this group experienced electrode failure, one directly due to wire breakage and one due to electrode displacement, confirming higher success rates with intramuscular electrodes. A recent innovation, the flexible epineural strip electrode (FLESE) [48], is made of polyimide as body material, along with three sensing electrode metal contacts coated with carbon nanotube. There are two different designs: the concentric bipolar electrode and the hook electrode. The hook electrode showed good adhesion properties sufficient to implant without suturing during acute recording in a rat experiment [48].

**Fig. 3:**
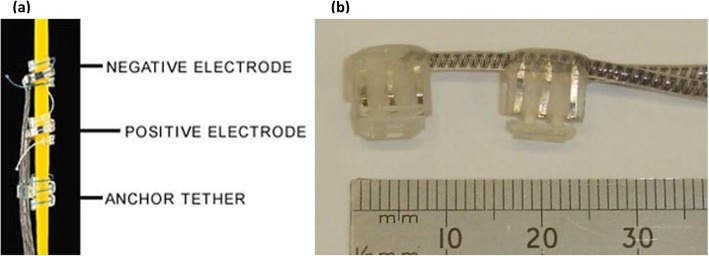
Two types of extraneural electrodes **a** Helical electrode [49] **b** Book electrodes [50]

The second type of extraneural electrode, the helical electrode (Fig. 3a), is created to wrap around the nerve, interfacing with one or multiple electrical contacts. It is composed of a Pt-Ir ribbon coil with an insulating layer of silicone, and MP35N alloy coils [51]. This electrode has been used for vagal nerve stimulation in order to treat intractable epilepsy and some forms of depression. It has been implanted in over 70000 patients [52]. Complications of this procedure include late infections, wound dystrophy, and rare incidents such as permanent vocal cord paralysis and twiddler’s syndrome, which involves the dislodgement of the electrode causing stimulation of unintended nerves [53].

Book electrodes (Fig. 3b) are well-known for their extensive use in sacral anterior root stimulation for bladder control. Each book electrode has three or five thin silicone rubber “pages” and each spinal nerve root is inserted into one of two or four slots between these pages; although other configurations have been proposed as well [54]. Each slot contains one cathode in the center and an anode at each of the two ends to avoid stimulation of tissue structures outside the slot [55]. Rhizotomy of the dorsal roots is common in practice during the implantation to improve continence. Absolute continence is often achieved after the procedure [56]. Reported serious complications include motor root damage and pain over the sacral dermatomes during micturition which can result in device abandonment [57].

Cuff electrodes are broadly researched as peripheral nerve interfaces. They are fabricated with various designs and are still continuing to be developed in advanced forms [58–66]. These designs are named and classified according to their morphological properties [10]. We will outline the types that have been proposed since it was first coined.

**Fig. 4:**
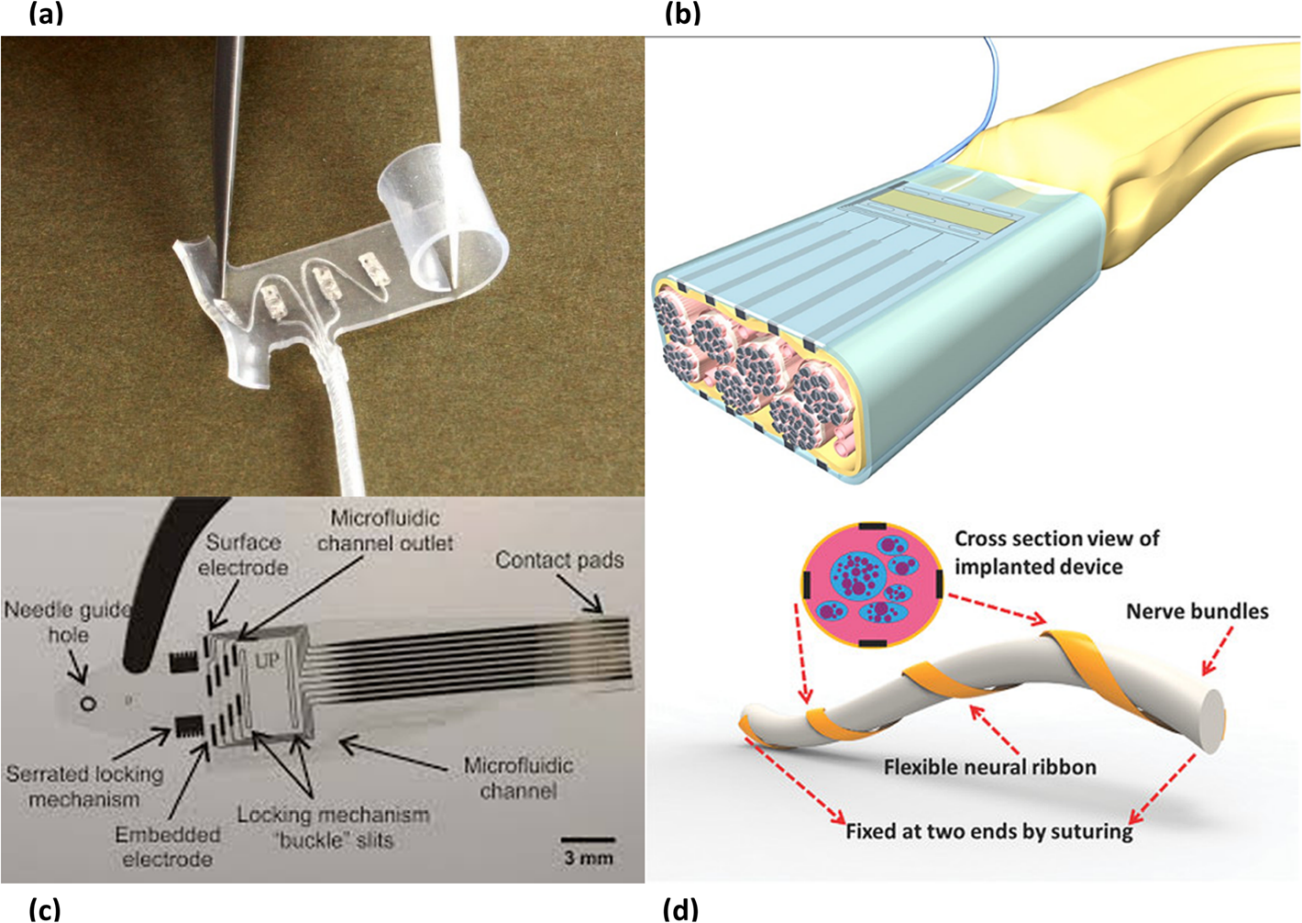
Different types of cuff electrodes **a** Spiral cuff electrode [68] **b** Schematic of the FINE (https://newatlas.com/renet-darpa/27750) **c** LACE [69] **d** Drawing of the neural ribbon electrode [70]

- The split cylinder cuff electrode is a cylindrical tube cut open longitudinally and placed around a nerve. It may be either sutured or installed without suturing [58]. The electrical contacts inside the tube may be concentric or longitudinal. The size of the electrode must be predetermined according to the target nerve [10], the inner diameter being slightly wider than the nerve. An interesting study conducted with pigs observed the outcomes of laparoscopic implantation of split cylinder cuff electrodes on pelvic nerves for 3 months [67], where the electrodes were functional at the end of the study. Moreover, the design was found to be easy-to-use for laparoscopic placement because it was not necessary to close the electrode once it was installed over the nerve.
- The spiral cuff electrode (Fig. 4a) comprises electrodes embedded within a self-curling sheath of insulation which exhibits a spiral transverse cross section. It is self-sizing and can accommodate diameter changes, for example, in case of neural swelling [59]. The inner edge of the cuff can penetrate the epineurium [71]. In a 3-year clinical study with two subjects, spiral cuff electrodes were shown to provide stable and selective stimulation without serious adverse reactions, after achieving stable stimulation thresholds around the 20^th^ week [72].
- The flat interface nerve electrode (FINE) is different from the aforementioned cuff electrodes in that it has a rectangular shape (Fig. 4b). It can have eight or twelve electrical contacts, half on the bottom and half on the top. It applies small forces to the nerve and reshapes it into a flattened oval form [60]. Thus, it effectively moves the axons closer to the electrical contacts, facilitating selective recording and stimulation. It is proven that FINE and the spiral cuff electrode, when used for stimulation of the afferent axons, produce stable and somatotopically selective results in human subjects in long-term applications, providing appropriate sensory information to the prosthesis wearer [73]. The newer version, the composite flat interface nerve electrode (C-FINE), minimizes the bulk and stiffness of the FINE, because it involves a stiff polymer bar laminated between two layers of flexible silicone sheeting [74]. The C-FINE was found successful in restoring sensation in individuals with a lower limb amputation [75].
- The flexible neural clip contains two gold electrodes coated with iridium oxide and it is designed for easy surgical implantation onto small nerves. The nerve can be inserted between the clip-strip and clip-springs after slightly bending the clip-springs [61]. It has shown good results in stimulating the pelvic nerve, the vagus nerve, and three branches of the sciatic nerve, as well as in the wireless stimulation of the pelvic nerve without implanting a power source [61].
- The flexible split ring electrode consists of a polyimide-metal-polyimide sandwich structure with four triangular bendable gold electrodes coated by carbon nanotube, fabricated with microelectromechanical systems technology [62]. It permits easy surgical implantation as in the case of the flexible neural clip. In a recent study [76], it enabled selective stimulation of the sciatic nerve, confirmed by the activations of the corresponding gastrocnemius and tibialis anterior muscles.
- Parylene-based cuff electrodes have an interlocking design eliminating the need for suturing or other fixing methods. The self-locking cuff electrode consists of a parylene strip with a guide tongue at the end and gold ratchet teeth along the edges, platinum microelectrode arrays, a gold locking loop, a parylene ribbon cable and pads for external connection [63]. Acute and chronic (11 weeks) implantation in rat sciatic nerve demonstrated its capability of selective stimulation. Another striking subtype is the lyse-and-attract cuff electrode (LACE) (Fig. 4c), fabricated from thin film parylene. It includes four surface microfluidic channels for localized delivery of lysing agents to disrupt the epineurium, followed by delivery of neurotrophic factors to enable axonal sprouting towards the pairs of platinum electrodes within each microfluidic channel [64].
- The neural ribbon electrode (Fig. 4d) is a polyimide-based stripe-like flexible device with eight electrical contacts, wrapped around the nerve only with fixed suturing at two ends [65]. It can be used for a wide range of nerve diameters owing to its flexible spiral nature.
- The nano-clip is fabricated using a direct-write 3D lithography system. It consists of two trapdoors with a semi-cylindrical interior channel, which permits smooth surgical implantation, and includes three holes for carbon nanotube electrode integration [66]. It demonstrated successful recording and stimulation in the tracheal syringeal nerve of the zebra finch in an acute trial [66].

Durability of cuff electrodes after implantation has been studied. Several failure modes have been reported including lead breakage, closing site failure, motion artifacts, and connection failure [77].

#### Interfascicular electrodes

In a nerve with multiple fascicles, groups of motor neurons are scattered among fascicles. These fascicles have different locations in the nerve, therefore their selective stimulation may be achieved by electrodes in their neighborhood [78]. Inter-fascicular electrodes were developed to guard the integrity of the fascicles by not penetrating them, but also to have a closer location to these fascicles than the extraneural electrodes.

**Fig. 5:**
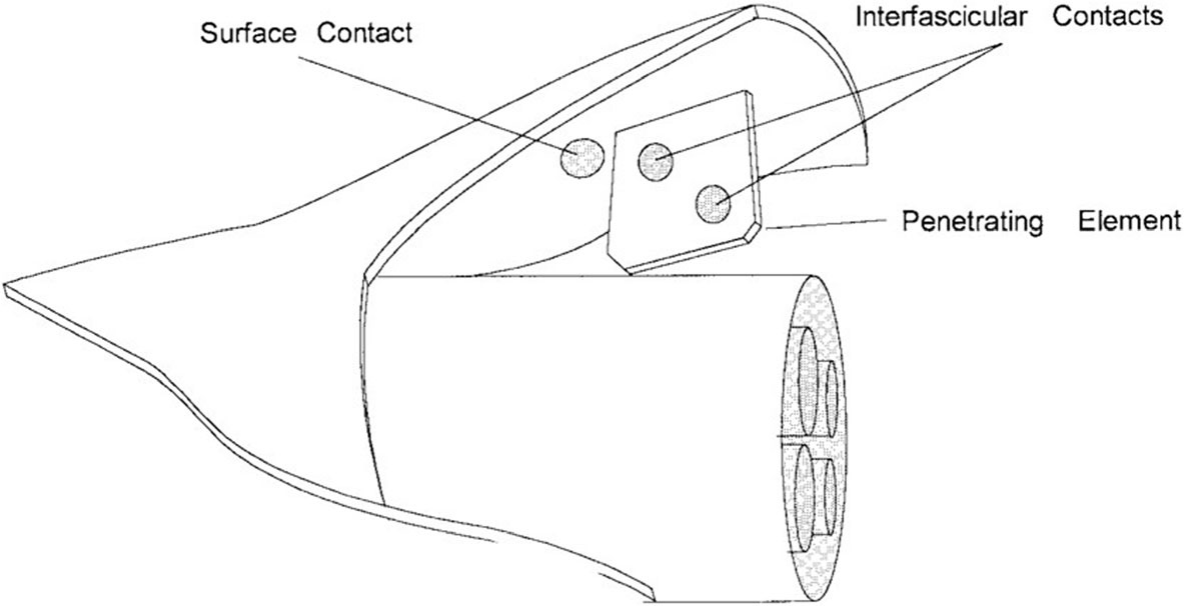
Schematic of the SPINE [79]

To be able to implant electrical contacts within the nerve, but outside the fascicles, the slowly penetrating interfascicular nerve electrode (SPINE) was invented. The SPINE applies small forces to the epineurium with blunt penetrating elements (Fig. 5) so that the epineurium is separated but the integrity of the fascicles are preserved. It comprises a silicon rubber tube with blunt elements in the lumen. Following implantation, the elements slowly penetrate within the epineurium without the interference of the surgeon [79]. In an acute cat experiment, the SPINE was implanted in the sciatic nerve and it has shown selective axonal recruitment, providing advantage over extraneural electrodes. Another result was that different interfascicular positions generated different recruitment, therefore placing contacts throughout the nerve will be beneficial in enhancing selectivity [79].

A distinct type of interfascicular electrode is the multigroove electrode which implements the opportunity of inserting different fascicles in different grooves. It is constructed on a teflon mold and is composed of multiple grooves each containing an electrical contact made of platinum-iridium alloy which is secured with silicone rubber. In an acute rat experiment, it has selectively stimulated one nerve fascicle without stimulating other neighboring fascicles [80]. The multigroove electrode has also shown success in minimizing muscle fatigue, as it provides the possibility of sequential stimulation of the fascicles instead of synchronous stimulation [81].

Two other designs which are similar to each other were developed and tested in rabbit experiments for selective stimulation and recording [82, 83]. The electrodes consisted of a flattened nylon tube with a nylon suture at one end for pulling the electrode into the nerve, and circular silver contacts installed in longitudinal pairs on each side of the nylon tube. One of the designs had 4 electrical contacts, 2 on each side; and the other design had 6 electrical contacts, 3 on each side. These studies also confirmed the capability of interfascicular electrodes in interfacing with subpopulations of nerve fibers. Nevertheless, the experiments concerning the interfascicular electrodes are all acute animal studies. Accordingly, the stability and reliability of these electrodes are yet to be discovered.

#### Intra-fascicular Electrodes

Another method to interface with the PNS is with intra-fascicular electrodes. While this method is recognized for its spatial resolution and selectivity, it is invasive with potential to damage the nerve. There are two main types of intra-fascicular electrodes based on their method of attachment: the longitudinal intra-fascicular electrode (LIFE), and the transverse intra-fascicular multichannel electrode (TIME). Penetrating microelectrode arrays (MEAs) could be considered a third type since they encompass an array of many tiny electrodes organized in an orderly framework; withal they are also attached in a transverse mode.

**Fig. 6:**
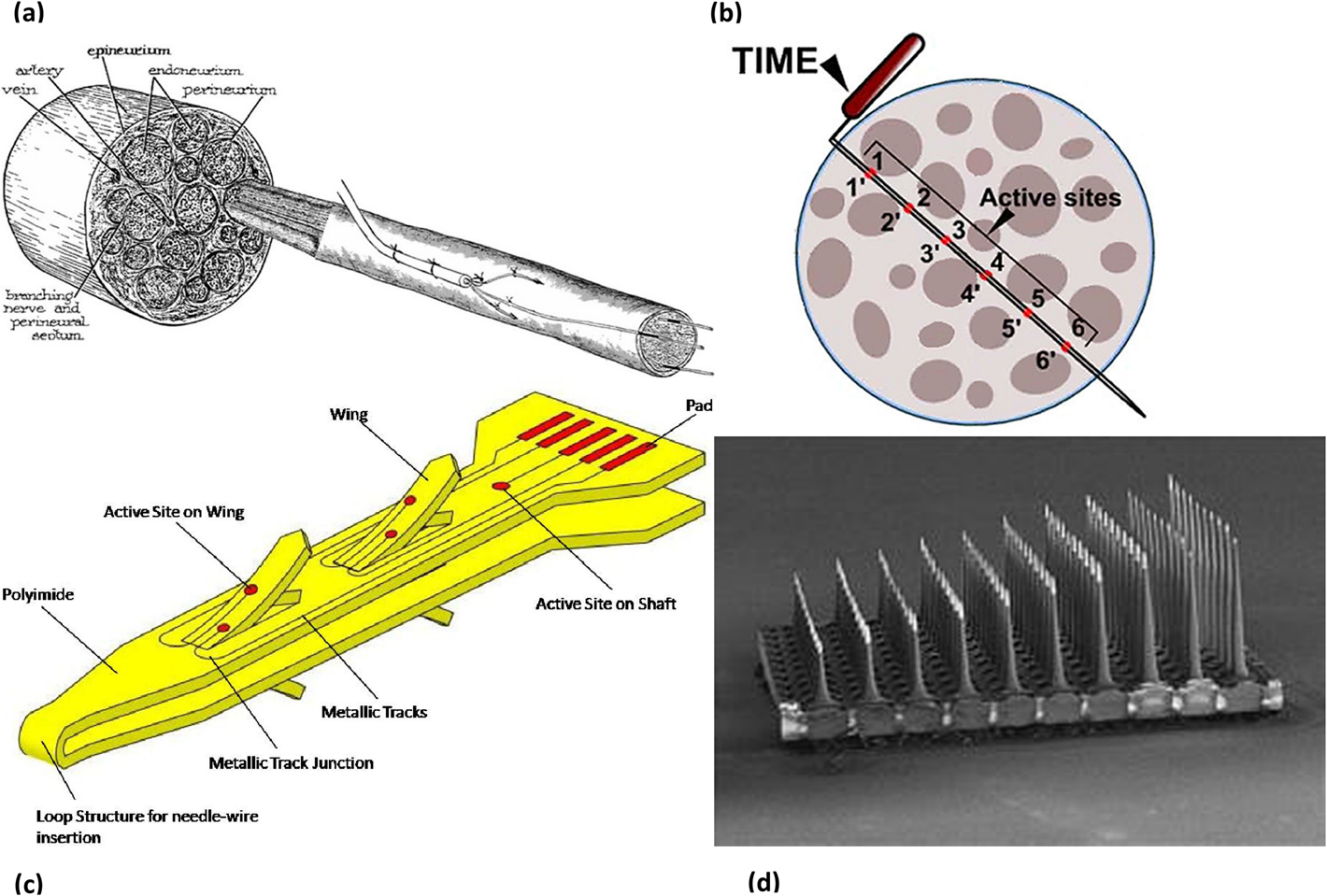
Several forms of intrafascicular electrodes **a** The intrafascicular drawing of LIFE [84] **b** Schematic of the TIME [8] **c** Illustration of the SELINE [85] **d** USEA [86]

The LIFE (Fig. 6a) is constructed from platinum or platinum iridium wires insulated with teflon, or from metalized Kevlar fibers insulated with silicone. It is placed longitudinally inside a nerve fascicle, and its small deinsulated region touches the nerve fibers [8]. It has been proved that LIFEs can sensitively record sensory signals [87]. They are also able to selectively stimulate nerve fibers, even enabling the activation of separate axonal subsets within a single fascicle [88], and the induction of graded responses [89]. A more advanced version of the LIFE, the thin-film LIFE (tf-LIFE), is a polyimide-based device constructed by a micromachining process, containing eight electrical contacts instead of a single one, and enables transverse implantation to interface with multiple fascicles. Although, longitudinally implanted tf-LIFEs showed less functional decline compared to transversally implanted ones during in vivo testing [90]. In a rat study, it caused a mild functional decline during the first month, attributable to nerve damage during implantation. Nonetheless the rats recovered after the following two months without any sign of nerve degeneration [90]. It has been implanted in clinical trials, and has demonstrated good performance with prosthetic arms. Multiple electrodes were implanted in different nerves and the amputees improved their control with training [91, 92]. Furthermore, the distributed intra-fascicular multielectrode (DIME), an evolution of LIFE, is designed in order to access multiple fascicles within a nerve. For the aim of providing more selectivity, it is composed of multiple LIFEs with their proximal ends coiled in a sheath to provide robustness and facilitate implantation [93]. Another study proposed a 15-electrode array lead that trifurcates into three groups of five LIFEs, and showed that it had sufficient mechanical fatigue resistance. Each group of LIFEs can be implanted in different nerves, and each LIFE can be implanted in different fascicles or in the separate areas of the same fascicle [94]. A 6-month rat study showed electrode breakage in a small percent of the electrodes leading to failure [95].

Designed as a hybrid of the tf-LIFE concept and selective microneurography approaches, the TIME comprises a thin strip-like polyimide substrate with multiple platinum electrodes. It is inserted transversely into a nerve and has an electrical contact interface with a different subset of axons (Fig. 6b). In the first acute experiment with rats, it allowed for considerable selective stimulation [96]. In a pig study comparing the selectivity of tf-LIFE and TIME, TIME was found to have a more complex recruitment pattern and was able to recruit a higher number of muscles with higher selectivity [97]. As in the case of tf-LIFE [90], TIME causes a time-limited damage in the nerve fibers and consequent axonal degeneration during the early phase post-implantation, as described in a 2 months rat study [98]. A recent clinical study with two transradial amputees has proven the capability of TIME electrodes in improving sensory feedback when combined with non-invasive somatotopic neural stimulation [99]. In another recent study, TIME electrodes were implanted in the median and ulnar nerves of a transradial amputee. The electrode in the median nerve was implanted for the purpose of stimulating afferent axons, and texture discrimination ability of the palmar surface of the index finger was reestablished for surfaces with constant and slow-sliding velocity [100]. In another real-time experiment performed with a dexterous prosthetic hand, the TIME electrodes were implanted in the median and ulnar nerves of an amputee. Touch sensation was restored to near-natural, thereby providing precise closed-loop control of the prosthesis [101]. In regard to lower limb amputees, a recent trial has documented the qualification of TIME electrodes in improving functional abilities, cognitive ease in dual-tasks, and prosthesis embodiment [102]. Each of the three transfemoral amputees was implanted with four TIME electrodes in the tibial nerve. They were equipped with a custom-made transfemoral prosthesis including a microprocessor-controlled knee with a knee encoder, and a sensorized insole. Tactile and proprioceptive sensations were reestablished, similar to a healthy extremity. These amputees were assessed in several functional tests, including ascending and descending stairs, walking on a field with obstacles with glasses impairing inferior vision, an auditory oddball task where the amputees try to count the target sounds while walking, and embodiment tests. Their functional capacity significantly improved after regaining touch sensation from the insole and proprioception from the knee joint, and they showed enhanced attention levels in the dual-task. TIME electrode implantation also resulted in increased embodiment of the prosthesis. Another clinical trial with two transfemoral amputees has shown that sensory feedback via TIME electrodes ameliorates walking speed, and decreases oxygen and energy consumption, which produces a lower cardiorespiratory burden [103]. Still, infection from the implants and external connector breakage have been reported as failure causes in a chronic study [104].

In terms of stability, a more advanced version of intra-fascicular interface has been proposed. The self-opening intra-fascicular neural interface (SELINE), a variation of the TIME, is a polyimide-based device which consists of a main body incorporating four lateral wings, and has two electrical contacts on each wing and one electrical contact on the main body (Fig. 6c). It is implanted by piercing the nerve with a needle that serves as a guide, followed by transversal insertion across the nerve and then a part inside the device is pulled back so that the wings open [85]. In a rat study comparing the stability and stimulation selectivity of the SELINE and TIME, SELINE was found to stimulate more neural subfascicles, and its stability indices were higher than the stability indices of TIME, validating the impact of open wings in preventing displacement [105].

Another method of intra-fascicular interfacing is using a penetrating MEA inserted transversely into a peripheral nerve. MEAs consist of a plane with numerous tiny electrodes constructed from various metals, enabling recording from and stimulation of a wide range of space in both the central and the PNS. Several types have been proposed [106–108], including MEAs for extracellular recording [109]. The Utah Slanted Electrode Array (USEA) incorporates 100 independent microelectrodes, separated by 400 micrometers on a 10 by 10 grid (Fig. 6d). A single USEA covers nearly all the depth and width of a cat sciatic nerve, staying in relation with a huge number of fibers [110]. An even higher density form (HD-USEA) in which the microelectrodes were separated by 200 micrometers was proposed for sub-millimeter neuroanatomical structures [111]. In a study done with four monkeys, the USEA implantation on elbow level and shoulder level promoted precise muscle activation. In particular, the extrinsic muscles of the hand were activated selectively, whereas the intrinsic muscles grouped for similar functions were often activated together [112]. In another study with two upper extremity amputees, the USEA was inserted in the median nerve of one subject and in the ulnar nerve of the other. These two patients became capable of controlling a virtual prosthetic hand, even though the patient with the median nerve implant suffered from wire breakage in the majority of microelectrodes [113].

#### Regenerative electrodes

**Fig. 7:**
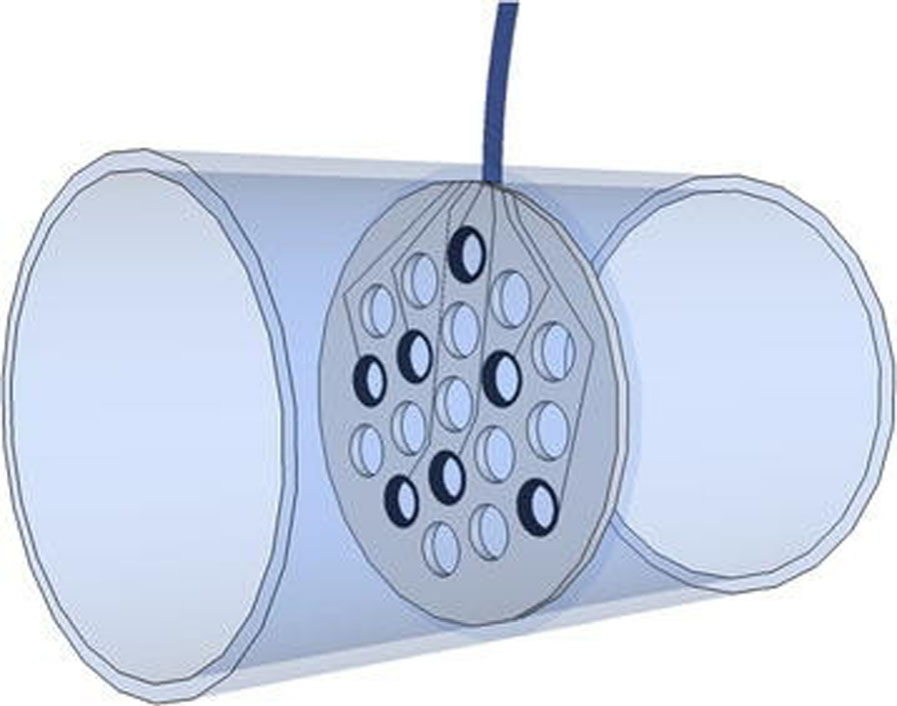
Schematic of a regenerative sieve electrode [115]

The last electrical interfacing method with the PNS is the regenerative electrode, which is devised to stimulate the transected nerve fibers to regenerate along numerous holes or channels. This type of electrode can have contact with a great number of axons. Various techniques have been proposed to induce nerve fiber growth across an electrode, such as the use of topographic cues, chemoattractants, material coatings, and biologics [114]. The first form, the traditional sieve electrode (Fig. 7) was designed 45 years ago and is a flat surface drilled through teflon-coated metal wires to open channels for the nerve fibers to grow through [116]. More advanced versions were constructed using flexible materials [117–120]. A recent design incorporated 64 channels in a double-layered sieve electrode in order to double interconnection density and increase spatial resolution. It was implanted in the rat sciatic nerve. The results were not favorable after 4 months in terms of motor function, which was attributed to inadequate porosity [121]. Polyimide-based sieve electrodes were investigated chronically in a 12-month study. Two types of electrodes, one with 281 holes and another one with 571 holes, were implanted in the sciatic nerve of rats. The second electrode was designed to increase the total surface area [118]. The percentage of regeneration was maximum by 6 months. Stimulation and recording of discrete axonal populations, both motor and sensory, were feasible. Signal amplitudes obtained by the second electrode were mildly higher. However, a minor decrease in the degree of functional reinnervation caused by compressive axonopathy occurred between 6 to 12 months, particularly in the distal segments. Another device called the regenerative scaffold electrode, which integrates a thin-film electrode array within a nanofiber sheet, was designed to minimally obstruct the area available for axonal regeneration. It was capable of recording neural activity after implantation in tibial nerve gaps of rats [122]. A newer model, the microchannel-based regenerative scaffold, incorporates top and bottom polydimethylsiloxane layers with SU-8 walls creating contiguous microchannels, which is rolled onto itself before implantation to form microchannel conduits. It was implanted in the sciatic nerve of rat amputee models, and the signals were obtainable for five months after implantation [120]. Several disadvantages of the rolling approach were defined by the authors, including inequality of the area of the microchannels, and a small open core forming in the center of the scaffold, causing the fibers to regenerate through itself instead of the microchannels. The regenerative multielectrode array, which is a MEA inside a regenerative tube, demonstrated good results after interfacing with chronically amputated nerves in a rat study. The axons grew through the array and the nerve signals were obtained as early as 1 week post-operatively, with increasing signal amplitudes as more electrodes were becoming active within the first 3 weeks [123]. The most common complications were wire breakage and infection. Moreover, the electrode-nerve interface showed limited inflammation in this acute experiment [123], proving the need for long-term studies. Of note is the double-aisle regenerative electrode which consists of a double-sided thin-film polyimide planar electrode, called the “septum”, with 12 circular active sides on each side, longitudinally inserted inside a silicone tube, creating two electrically-insulated compartments to allow regeneration of separated nerve fascicles [124]. For experimentation, tibial and fibular nerves of the rat were chosen for each aisle. Selective stimulation and recording of the fascicles were possible both acutely and chronically, with a slight decrease in selectivity after 3 months. The number of “septa” can be increased to further increase compartmentalization, and the control of the population of axons in each compartment (for example, motor neurons to ankle evertors in one aisle, sensory neurons which innervate the skin over the posterolateral part of the leg in another aisle) seems possible by incorporating specific neurotrophic factors in chosen compartments.

### Non-electrical methods

#### Mechanomyography

Mechanomyography (MMG), or vibromyography (phonomyography), is a non-invasive technique to measure lateral oscillations provoked by muscle contraction and stretching, which can be recorded by a hydrophone, piezoelectric contact sensor, a laser distance sensor, a microphone or an accelerometer [125]. It is not affected by skin conditions as opposed to non-invasive surface electrodes [126], although it is more sensitive to muscular fatigue [127]. In a study done with able-bodied subjects, it was found superior to EMG in processing signals from hand muscles [125]. As in all methods that interface with the muscles but not the nervous system itself, MMG constitutes an indirect peripheral nerve interface.

#### Magnetoneurography

Magnetoneurography (MNG) is a method to directly measure the conduction capacity of a nerve by recording the biomagnetic action fields, which are hardly influenced by the impedance of the surrounding tissues [128]. It has wide applications in the diagnosis of peripheral nerve disorders [129]. It can also be used to record signals from nerves for the intent of neuroprosthetic control; however the fact that the measurements are done in a magnetically shielded room restricts its use [12].

#### Optogenetics

Optogenetics comprises the insertion of opsin proteins, which are light-sensitive ion channels that convert photons to electrochemical signals, into living cells. Thus, the motor neurons can be genetically modified to express opsins so that they can be activated and inhibited by light, which results in muscle control. In fact, optical stimulation promotes the ability to orderly recruit the muscle fibers, meaning that the slower motor units can be activated first [130]. Optical stimulation of the sensory neurons is also attainable, and the induction of sensation is tunable, meaning that the intensity of light can be adjusted in order to achieve the optimal level of excitation or inhibition [131]. The incorporation of the opsins can be achieved via transgenic approaches, viral vectors, or by targeting axonal projections via retrograde transport; owing to these techniques, specific neuronal subpopulations can be selectively activated [132]. The studies concerning neuronal excitation and inhibition were trying to achieve co-expression of an excitatory opsin such as channelrhodopsin-2, and an inhibitory opsin such as halorhodopsin. A study done with mice proved that both excitation and inhibition can be obtained through using only channelrhodopsin-2 [133]. A recent study observed the effects of engrafting embryonic stem cell-derived motor neurons expressing channelrhodopsin-2, into a denervated peripheral nerve. The results demonstrated that regenerative medicine and optic stimulation can be used together to reinnervate peripheral nerves [134]. The outcomes of the application of optogenetic methods in the control of neuroprosthetic limbs, however, are yet to be discovered.

#### Focused ultrasound

This non-invasive method can be used to modify activity of the nerve cells and affect their conduction properties [135]. Focused pulsed ultrasound has been utilized to excite and inhibit the vagus nerve, in pursuance of an alternative method to treat epilepsy and depression [136]. High-intensity focused ultrasound was also used to suppress nerve conduction in rat studies [137, 138]. Unfortunately, the specific parameters affecting the neuromodulation process, such as the extent and limits of thermally induced damage on nerves, have not been completely established. Accordingly, the usage of focused ultrasound for interfacing with peripheral nerves to control prosthetic limbs is waiting to be explored.

#### Infrared nerve stimulation

Pulsed infrared laser lights have also been used to stimulate neural activity in motor and sensory systems [139]. This technique combines the advantage of stimulation without contact and the relatively high spatial resolution, owing to the lack of current spread and stimulation artifacts [140]. On the other hand, the risk of thermal damage should be avoided as in the case of focused ultrasound. Recent studies with rats tried a hybrid approach, combining peripheral nerve electrodes and infrared nerve stimulation. This method yielded good results in generating sustained muscle contractions [141], and it demonstrated the decrease in required optical energy when infrared nerve stimulation was combined with electrical methods [142]. Future studies applying the combination of these techniques for the control of prosthetic limbs can produce favorable results.

## Interface design considerations

There are critical aspects of an interface design that influence the harmony between the patient and the interface. These design considerations are emphasized in this section, including the device biocompatibility, its selectivity, the transfer of signals to and from the external hardware, and long-term reliability. This section and the following sections will focus on electrical methods to interface with the PNS.

### Biocompatibility

Any implantable medical device must be evaluated for its biocompatibility, which can take many forms. In the context of electrode interfaces, biocompatibility involves any undesirable effect that the device can cause and any undesirable tissue response. One of the fundamental standards is the ISO 10993 series. Electrically active medical devices should be tested for electromagnetic compatibility, which involves the device’s susceptibility to electromagnetic interference from any source.

The possible tissue reactions include blood-material interactions, provisional matrix formation, inflammation, granulation tissue formation, foreign body reaction, and fibrosis [143]. Hypersensitivity reactions might occur in specific patients. Choosing the appropriate interfacing method depends on the precise needs of the patient, as the degree of spatial resolution and selectivity of the interface usually increases with its invasiveness. Lead migration, fracture, and malfunction; wound infection and breakdown; hardware failure and removal are some of the most common complications related to interface design [144].

Peripheral nerve implants can also result in peripheral nerve injury. This can lead to Wallerian degeneration, neuropathic pain, and fibrosis, resulting in signal loss [14]. Therefore, adequate attention must be paid to comply with the properties of the nervous tissue when designing an interface. General procedures to minimize the tissue response include coating implantable devices with anti-inflammatories, surface modification with polyethylene glycol, and electrode site modification [145]. The risk of microbiological colonization of the implant should also be taken into account. Established techniques are dry heat sterilization, steam sterilization, ethylene oxide sterilization, radiation sterilization, hydrogen peroxide sterilization and ozone sterilization [146].

In regard to optogenetic approaches, there are critical challenges to consider when designing a biocompatible interface. First, the adaptive immune system of the subject can produce antibodies against the transgene or against the capsid antigens of the viral vector. Second, as is the case of all gene therapy modalities, there is a risk of oncogenesis [147]. The risk of photodamage should also be evaluated. Red light has been shown to induce less marginal phototoxicity than blue light, therefore red light-activated opsins such as VChR1, ReaChR and Chrimson variants can be utilized [148]. Moreover, the stimulation parameters of optogenetic applications, focused ultrasound, and infrared nerve stimulation should be adjusted in order to prevent heat-induced tissue damage. High intensity focused ultrasound induces irreversible cell death [149], and low intensities are unable to evoke action potentials in peripheral nerves, unlike in the neurons of the brain. Thus, intermediate intensity range (1 – 200 W/cm^2^) should be employed for safety [150].

### Selectivity

The second important property of an interface is its capability of selective stimulation and its spatial resolution; in other words, it should be minimally disturbed by the surrounding tissues and maximally communicating with the target. The possibility of decrease in spatial resolution in vivo due to inflammatory tissue response should also be considered [151].

Intra-fascicular electrodes are expected to selectively stimulate a more concentrated group of nerve fibers since they are closer to the target. In addition, extraneural electrodes can generate an electrical field when used in multiple quantities and thus can offer increased selectivity superior to single electrode implantation [152]. Choosing FINEs as extraneural interfaces can also increase the spatial resolution in comparison to spiral cuff electrodes, as it modifies the shape of the nerve to become adjacent to the fascicles [153]. Furthermore, multi-contact cuff electrodes are also able to selectively recruit a single muscle [154]. In a study comparing the multipolar cuff electrode, LIFE, and TIME, TIME was found to be the only electrode to selectively stimulate at both interfascicular and intra-fascicular levels, whereas LIFE was stimulating selectively at the intra-fascicular level and cuff electrode was allowing selectivity in interfascicular level [155].

### Transfer mechanism

**Fig. 8:**
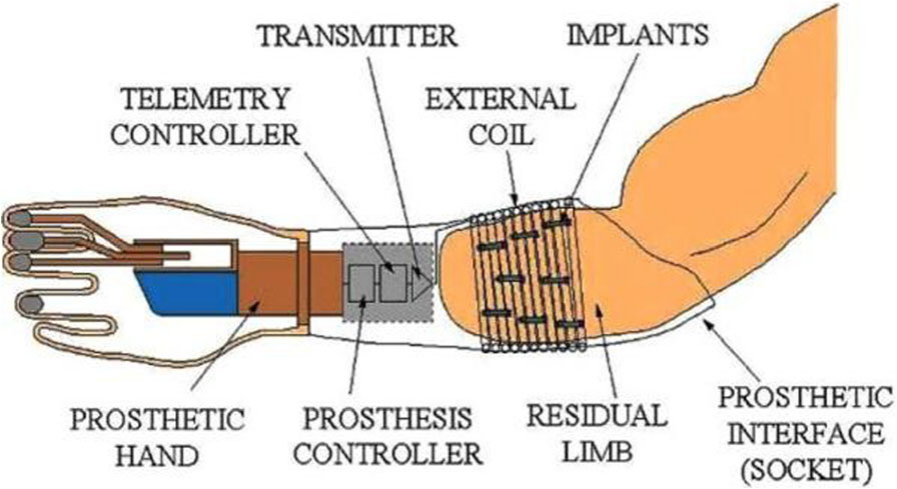
Schematic of IMES communication [158]

A further aspect to take into account is the transfer of signals and data to and from the external hardware. Wired electrical connection via the percutaneous route can be used for implantable electrical interfaces. However, wireless communication is more suitable for daily life, and is more beneficial in terms of patient motivation. The wireless chips should be packaged with nonconductive materials, therefore it is hard to achieve actively operated wireless communication systems. Moreover, power management is more difficult as implanted stimulators have one-time use batteries [156]. To overcome this issue, energy harvesting, which promotes the use of energy derived from the internal sources of the body, or wireless power delivery strategies can be exploited [157]. The IMES system (Fig. 8) was invented to avoid the disadvantages of percutaneous connectors. It consists of up to 32 implantable myoelectric sensors, an external power coil and receiving antenna, and a telemetry controller which passes data from the implants directly to the prosthesis controller [158]. Except for the sensors which are fixed in the muscles of the residual limb, all of the components are placed in the prosthetic socket. The sensors are small, cylindrical electrodes and are inserted into multiple muscles; therefore individual interfacing with each muscle can be accomplished. This system is originally intended for the upper extremity and the ongoing trials are carried out with upper extremity amputees. The chronic first-in-man demonstration was in a right transradial amputee performed 11 months following amputation. Eight electrodes were placed in different muscles and the patient was asked to limit movement to prevent migration of the sensors. Three weeks after implantation, he was able to operate the prosthesis immediately with 3 degrees of freedom [159].

As for optogenetic stimulation, a fully implantable optoelectronic device which contains its own light source with a stretchable antenna that harvests wireless radio power has been developed, and it has shown promising results when tested to evoke pain sensation in mice [160]. A further fully implantable, battery-free, wireless optogenetic device equipped with tiny LEDs powered by resonant magnetic coupling has demonstrated its ability to excite opsin-expressing nociceptors in mice, and it can be operated by a smartphone [161].

It is also important to highlight the concept of osseointegration in this section. Osseointegration is the direct anchorage of a metal implant into living bone, creating a functionally and structurally stable connection between biological tissue and synthetic material [162]. The preferred metal is titanium, as an extensive oxide layer forms outside of it when it is subject to bone tissue, which strengthens the anchor. When used in amputees, osseointegrated implants eliminate the problems associated with socket prostheses since these implants are continuous with an abutment which penetrates the skin and serves as an attachment point for the prosthetic limb [163]. A major benefit of fixation via osseointegration is the prevention of reliability problems associated with percutaneous leads [164]. Another benefit is osseoperception, which can be defined as the ability of patients with osseointegrated implants to receive tactile sensory information via mechanical transmission through their prostheses [165]. In a clinical study in which a subject was followed up for 25 months post-operatively, osseointegration has been shown to provide localized sensory inputs when combined with cuff electrodes [166]. This demonstrates the competence of this transfer mechanism in the field of closed-loop neuroprosthetics.

### Longevity

Finally, interfacing with the PNS for prosthetic limb control is a chronic circumstance and the long-term outcomes and functionality depend highly on the stability and the reliability of the interface being used. Some of the common adverse events that hinder the longevity of the implanted interfaces are micro-motion, wire breakage, delamination, and insulation breakdown. The patients should be regularly monitored in order to be aware of any adverse event and the appropriate action should be taken. Implanted interfaces should be radio-opaque, to allow follow up with radiographs [167].

## Fabrication technologies

There have been numerous improvements in the fabrication technology of neuroprosthetic interfaces. One of the notable advancements is the utilization of nanostructured systems at the neural interface. As the cellular environment of the neural tissue is composed of elements of several nanometers, nanoscale surface modification of the implanted systems can mitigate the challenges related to mechanical properties and inflammatory response [168]. Nanomaterials such as carbon nanotubes or graphene have been used to construct interfaces with the central nervous system too [169].

Another application in the production is tissue engineering the neural interface. By integrating a layer of encapsulated neural cells in the electrode coating, the mechanical issues arising from the stiff properties of the conductive materials can be eliminated and thus the formation of scar tissue can be decreased [170]. This concept of “living electrodes”, however, has yet to be tested in vivo.

Although not applicable for neuroprosthetic limb control, a recent advance in the field of peripheral nerve interfaces is the creation of bioresorbable electrodes composed of elements that the body can metabolize [171]. These implants have the capability of recording and stimulation for a short duration. They can be used in the treatment of various nerve injuries [172], but not for interfacing with prostheses.

## Long-term outcomes

As indicated in the last section, it is obvious that an amputee will need a long-term interface to control a neuroprosthetic limb. Longevity of the implanted interfaces, as determined by chronic in vivo studies, are discussed in this section. Table 1 summarizes the results of the chronic in vivo studies in humans. Table 2 summarizes the results of the chronic in vivo studies in animals, using the LIFE, SELINE and regenerative electrodes, due to the lack of long-term human studies with those interfaces.

**Table 1:** Summary of the results of the chronic in vivo studies in humans

Interface	Longevity
Muscle electrodes	Stable after 16 years [173]
TMR combined with IMES	Stable after 2,5 years [174]
Epineural electrode	Stable after 18 years [175]
Cuff electrode	Stable after 11 years [176]
TIME	Stable after 6 months [177]
USEA	Stable after 14 months [178]

**Table 2:** Summary of the results of the chronic in vivo studies in animals, with the LIFE, SELINE and regenerative electrodes

Interface	Longevity
LIFE	A life-time of 18-24 months is expected [179]; slight tissue damage in the early phase but normalizes after 6 months [180]
SELINE	Inflammatory response in the early phase, but stable after 6 months [181]
Regenerative electrodes	Inflammatory response to nerve transection, but good signal quality after 4 months [182]; another 12-month study observed regeneration and reliable signals in the first 6 months, but axonal damage and functional decline in the second 6 months [183]

In a clinical study with 27 patients, 238 muscle electrodes, both epimysial and intramuscular, were implanted and followed up for 16 years. The failure rate was less than 2 percent. Only three lead failures occurred and one localized infection took place at an electrode, which leads to the conclusion that muscle electrodes can be used confidently for long-term applications [173]. A recent prospective study conducted with three above-elbow amputees observed for two and a half years clearly demonstrates the functional outcomes of TMR surgery combined with IMES implantation [174], where the subjects attained intuitive and reliable control of their prostheses. In another experiment conducted with monkeys, nine RPNIs were combined with epimysial electrodes and the monkeys were monitored for 14 months. Independent signals from each RPNI could not be evaluated, but stable prosthesis control was achieved [184]. A major issue with myoelectric prostheses is the intensive training regimen required to effectively operate the device. Approximately 30% of the patients abandon their prostheses before mastering control [185].

The longest study with nerve electrodes that we could find was an 18-year clinical study with 12 quadriplegic patients which observed the outcomes of a specific form of epineural electrode implantation on phrenic nerve to restore ventilatory function [175]; most of the patients were unable to continue the study owing to the co-existing medical conditions, but these electrodes were found to be biocompatible and safe to use in chronic applications. In an 11-year clinical study which analyzed the results of spiral cuff electrode implantation in 14 different patients. The electrodes were functional, selective, and free of serious adverse events after 11 years of follow up [176]. In another study, an implanted stimulation generator composed of epimysial and cuff electrodes was implanted in a paraplegic patient to reestablish lower limb motor function and the patient was followed for 9 years. A few months after implantation, the patient was able to walk for 30 minutes 5 to 10 times per day, and neural stimulation was found to be more stable, with less adverse events, but muscle fatigue remained the main issue in both electrode types, marking the importance of muscle strength training [186]. The adverse events caused by epimysial electrodes were presumably due to the stimulation of the neighboring afferent nerve fibers, as reported by the authors.

The experiments concerning intra-fascicular electrodes are relatively of short duration. LIFEs were evaluated in a 6-month cat experiment. The electrodes produced signals of good quality at the beginning, but there was a slight decrease during 6 months. The authors expect a functional lifetime of 18 to 24 months if the same decrease in quality continues [179]. Polymer-based LIFEs were also found to be biocompatible during a 6-month study with rabbits, with slight connective tissue buildup but minimal electrode drift [187]. A further 9-month study done with rabbits observed a tissue damage after LIFE implantation, leading to impaired signals in the first two months, but this decline started to normalize after the third month and fully normalized after the sixth month [180]. A rat study demonstrated the recording and stimulation capabilities of tf-LIFE for three months. There was a functional decline but it was reversible and the electrodes were operational at the end of three months, as in the case of aforementioned study [90]. Likewise, the good performance of tf-LIFE after 3 months was demonstrated in a human volunteer, where three different hand movements were accomplished [188]. A different study evaluated the outcomes of TIME implantation in the median nerve of pigs during a follow up of 38 days. There was no necrosis, the formation of fibrotic capsule was limited, and all the electrodes were stable [189]. More recent studies with transradial amputees have shown the feasibility of TIME implantation in the median and ulnar nerves for 6 months [104, 177]. The electrodes used in this study were polyimide-based and had silicon carbide adhesion layers which contributed to their stability. A study with TIME electrodes showed positive effects of systemic dexamethasone administration on decreasing foreign body reaction, further ensuring the chronic functionality [190]. A further study evaluated the results of SELINE insertion into the sciatic nerve of rats for 6 months [181]. Inflammatory response with swollen fibers was observed in the early phase after implantation, but it gradually resulted in capsule formation around the place of implantation, and all of the electrodes were functional at the end of the study.

Penetrating microelectrodes confer huge spatial resolution and selectivity in acute studies, enabling the stimulation of particular wrist movements such as flexion or extension, and allowing the recording of specific finger movements when grouped in subsets [191]. A chronic study which evaluated the results of USEA implantation to the sciatic nerve of cats for 350 days and showed long-term presence of a USEA in the sciatic nerve caused persistent inflammation that extended until the end of the study, and a decrease in fiber diameters, without a significant change in the degree of myelination [151]. On the other hand, a recent trial with a transradial amputee combined EMG signals with two USEAs for 14 months [178] demonstrated reliable recordings during the entire study. The influence of improved tactile perception and object discrimination with these electrodes on dexterous prosthetic control with greater patient motivation has been proven.

A 3-month study conducted with rats observed the functionality of a regenerative macro-sieve electrode. The electrode enabled axonal regeneration, and the stimulation of different electrode sites recruited specific muscles with a continuing improvement [192]. Another rat study monitored the consequences of regenerative multielectrode interface implantation in the sciatic nerve after 4 months. There was a severe inflammatory response to nerve transection at first, but the signal-to-noise ratio was high enough for recording during 120 days [182]. A 12-month rat study examined the results of chronic polyimide-based regenerative sieve electrode implantation [183]. Axonal regeneration progressed during the first 6 months, but there was a functional decline in some of the electrodes during the second 6 months, with histological evidence of demyelination and axonal degeneration.

## Conclusions

In this review, we summarize both mature and novel techniques to interface with the PNS in order to achieve sustained control of a neuroprosthetic limb. A few of the interfaces had been used for decades in the treatment of nerve injuries, as well as for functional electrical stimulation applications. However, their use in the prosthetic field is much less enlightened. Chronic studies with muscle and nerve electrodes have shown great promise for a peripheral nerve interface. Further chronic studies evaluating long-term outcomes are needed for true appraisal of the long-term durability of the more invasive nerve electrodes. As technological progress provides new methods to design, fabricate, implement, and monitor advanced devices and applications, amputees will have the opportunity to attain long-term, reliable, intuitive control of their prosthetic extremities.

## Data Availability

Availability of data and material is not applicable to this article as no datasets were generated or analyzed during the current review.

## Abbreviations

C-FINE: Composite flat interface nerve electrode
DIME: Distributed intra-fascicular multielectrode
EMG: Electromyography
FINE: Flat interface nerve electrode
FLESE: Flexible epineural strip electrode
HD-USEA: High-density Utah slanted electrode array
IMES: Implantable myoelectric sensor
LACE: Lyse-and-attract cuff electrode
LIFE: Longitudinal intra-fascicular electrode
MEA: Microelectrode array
MMG: Mechanomyography
MNG: Magnetoneurography
PNS: Peripheral nervous system
RPNI: Regenerative peripheral nerve interface
SELINE: Self-opening intra-fascicular neural interface
SPINE: Slowly penetrating inter-fascicular nerve electrode
tf-LIFE: Thin-film longitudinal intra-fascicular electrode
TIME: Transverse intra-fascicular multichannel electrode
TMR: Targeted muscle reinnervation
USEA: Utah slanted electrode array
